# Identification of Fish Interferon-Stimulated Genes and Their Antiviral Mechanisms

**DOI:** 10.3390/v18030296

**Published:** 2026-02-28

**Authors:** Emily Yang

**Affiliations:** Department of Biological Sciences, Biola University, La Mirada, CA 90639, USA; emily.yang@biola.edu

**Keywords:** interferon-stimulated genes, teleost, IFIT1, Mx, Nmi, IFP35, Viperin, TRIM25, finTRIM, ISG15

## Abstract

The frontlines of innate antiviral immunity center on type I interferons (IFNs), which are expressed by nearly all cell types as a cellular alarm signal. IFNs drive the expression of IFN-stimulated genes (ISGs), which can both generate an intracellular antiviral state and regulate the IFN response itself. This key antiviral line of defense is conserved in all jawed vertebrates, including teleost fish. Since their identification nearly 70 years ago, many mammalian ISGs have been identified and characterized However, fish ISGs represent an exciting, largely unexplored avenue of antiviral effector research and present an opportunity to assess how IFN systems have been shaped by whole genome duplication events. This review summarizes advances in the identification of bona fide teleost ISGs and examines studies elucidating the antiviral mechanisms of conserved ISGs, including IFIT1, Mx, Nmi and IFP35, Viperin, TRIMs, and ISG15. Teleost-specific gene expansions and isoform divergence, particularly in the development of the fish novel TRIM family, will be considered under each relevant ISG. Understanding teleost ISG biology promises not only to improve antiviral strategies in aquaculture but also to reveal novel antiviral principles with translational relevance for human health.

## 1. Introduction

There is virtually no cellular organism exempt from the ravages of viral infection. Cells have evolved defense mechanisms, from the elegant prokaryotic CRISPR-Cas defenses to T cell and B cell manned adaptive immunity in higher vertebrates. In between these ends of the spectrum exists innate immunity, well exemplified by interferon (IFN) responses as a first line of intracellular defense against viruses.

Identified in 1957 by Isaacs and Lindenmann nearly 70 years ago [1], the IFN response was so named for its ability to “interfere” with viral replication. The IFN response is well-characterized in placental mammals, which possess four IFN types: type I IFN (IFN-α, IFN-β, IFN-δ, IFN-ε, IFN-ζ, IFN-κ, IFN-μ, IFN-ν, IFN-τ, and IFN-ω), type II IFN (γ), type III IFN (λ), and the recently identified type IV IFNs (υ) [2]. Type II IFN primarily serves as a regulatory cytokine in innate and adaptive immunity, while type I and III IFNs are the major innate antiviral cytokines and regulate overlapping sets of genes. Though Type I and III IFNs bind to different cellular receptors (IFNAR1/2 and IFNLR1/IL-10R2, respectively), they share the same intracellular JAK-STAT signaling pathway (reviewed in [3]). Moreover, type I IFN receptors exist on almost all nucleated cells while IFNLR1 expression is restricted primarily to epithelial tissues [4].

Many excellent reviews on type I IFN signaling exist [5,6]. Before type I IFN signaling can be activated, type I IFN expression must first be induced. Briefly, upon sensing of pathogen-associated molecular patterns, such as cytoplasmic double-stranded RNA, pattern recognition receptors such as RIG-I-liked receptors are activated and induce an intracellular signaling cascade. Activated transcription factors drive the expression of type I IFNs, a secreted protein that then signals in both an autocrine and paracrine fashion via binding to IFNAR1/2. As mentioned previously, IFNAR1/2 signaling via the JAK/STAT pathway activates the ISGF3 transcription factor complex, which binds to the IFN-sensitive response element found in the promoters of hundreds of IFN-stimulated genes (ISGs). It is induction of these ISGs that directly creates an antiviral environment [3]. These ISGs are able to interfere with viral replication directly through many diverse mechanisms, such as shutting off global protein translation, targeting viral RNA for degradation, inhibiting viral capsid uncoating, acting as a pathogen sensor itself, and many more yet to be characterized mechanisms [7,8,9]. ISGs also often possess a regulatory function of the IFN response itself, serving as both positive regulators to potentiate the response or negative regulators to keep it from ballooning out of control [10,11,12].

While evidences of IFN-like systems in invertebrates exist [13,14,15], the innate IFN response is generally thought to have originated in gnathostomes, or jawed vertebrates [16,17,18,19]. Constituting more than half the vertebrates are the teleosts, the largest clade of ray-finned fish, which exhibit high biodiversity in response to the divergent ecological niches that they occupy [20]. Moreover, it is thought that a whole genome duplication (WGD) event occurred not only in the teleost fish common ancestor prior to diversification but also in specific teleost branches, such as the salmonids and cyprinids [21,22,23,24]. These WGD events offer up vast evolutionary potential for genes, allowing cells and organisms to adapt more freely to stressful environments as selective pressure on individual gene copies relaxes [25]. In fact, expanded gene families for several IFN signaling components and ISGs exist in fish that do not in higher vertebrates [26,27,28]. For example, fish possess a diversified type I IFN lineage, further subdivided in zebrafish into group 1 (φ1/φ4; two cysteine-containing) and group 2 (φ2/φ3; four cysteine-containing) IFNs [27].

The examination of teleost ISG antiviral mechanisms is a worthwhile endeavor for several reasons. First, as touched upon earlier, teleost fish possess an evolutionary potential unmatched by other vertebrates. Elucidation of their antiviral mechanisms may offer novel insights into ways mammalian ISGs can be re-engineered to be even more effective without the requirement of millions of years of evolutionary pressures. Second, fish represent an economically important source of sustenance; global production of animal-based seafood reached 185.4 million tons in 2022, with aquaculture contributing more than half of that supply at 94.4 million tons [29]. Aquatic animal foods are especially important in lower income countries. While they constitute about 15% of the animal protein supply worldwide, they reached over 50% in several Asian and African countries [29]. Despite its burgeoning prominence in food supply, aquaculture is especially vulnerable to viral pathogens that can cause hundreds of millions of dollars in economic losses, for which limited viable treatments exist [30,31,32]. Therefore, a more robust understanding of the way fish naturally defend themselves against viral infections may inform the design of novel antiviral therapeutics.

While in more recent years a number of other reviews have focused on specific teleost ISGs [33,34], the last review aimed at compiling a wide-ranging list of teleost ISGs was published in 2016 [28]. Additional reviews have focused on describing viral infections of fish and the transcriptional activation of several antiviral ISGs, but refrained from describing their antiviral mechanisms [35]. Therefore, this review aims to close the gap by examining the state of research of teleost ISGs that has not been comprehensively reviewed in the last five years at the time of writing this publication.

This review is limited to those ISGs identified in teleost fish and the ways in which they affect viral replication, which include direct antiviral activities as well as the regulation of the interferon response itself. Genes that affect the interferon response or viral replication without being conclusively established as an ISG (i.e., expressed in an IFN- or IFN receptor-independent manner) are excluded from the scope of this review. I will begin by first describing progress made in identifying ISGs within teleost fish. I will then discuss the mechanisms of conserved ISGs such as IFIT1, ISG15, Mx, Nmi and IFP35, and Viperin, before concluding with a discussion of ubiquitination and the ubiquitin-related genes TRIMs and ISG15. Throughout this discussion of ISGs, I will highlight teleost-specific gene duplications and diversifications that lead to divergent activity or expression.

## 2. Identifying ISGs: Cross-Referencing Conserved ISGs in Transcriptomics Studies and Whole Genome Assemblies

Identification of true teleost ISGs has proven to be challenging. Early work in the field focused on identifying orthologs to human ISGs [36], differential display PCR [37], or subtractive suppressive hybridization of cDNAs from poly(I:C)-stimulated or virally infected cells [38,39,40]. Poly(I:C) is a dsRNA mimic that strongly induces the type I IFN response. Genes upregulated by viral infection in fish were initially named *vig*, for “viral hemorrhagic septicemia virus (VHSV, -ssRNA)-induced gene”, and numbered in order of discovery [37,38,41]. Since then, advances in genome database availability have enabled the identification of mammalian orthologs for such virally induced genes and naming them likewise. More complete genome assemblies and advances in microarray analyses and RNA-sequencing enabled the identification of more virally induced genes [42,43,44,45,46,47,48]. These approaches identified genes that have a large degree of overlap with but are not necessarily ISGs, that is to say, IFN-dependent.

Pioneering work in identifying a conserved set of ISGs between mammals and zebrafish (*Danio rerio*), a model teleost and vertebrate organism, was performed by Levraud and colleagues in 2019 [49]. Here, deep RNA sequencing of zebrafish larvae was performed under a variety of conditions designed to refine a list of true zebrafish ISGs: direct inoculation with recombinant zebrafish type I IFN (IFNφ1), infection with the strong IFN-inducing chikungunya virus (CHIKV), and CHIKV infection when the IFNφ1 receptor had been knocked down via morpholino treatment. Direct IFNφ1 treatment identified more than 300 upregulated genes, 97 of which were orthologous to at least one human ISG, including the well-known antiviral effectors Mx, Viperin, IFIT, ISG15, and PKR [49]. Interestingly enough, 41% of these upregulated genes had no ortholog in the human genome, and many belonged to teleost-specific TRIM families, to be discussed in depth later in this review. All in all, the work here identified 72 ancestral genes maintained in both fish and tetrapods as part of a full list of zebrafish ISGs [49].

Studies endeavoring to identify teleost ISGs since then have relied largely on this zebrafish ISG list, cross-referencing genes identified in their own datasets in varied teleost species, such as the common carp (*Cyprinus carpio*), Atlantic salmon (*Salmo salar*), and rainbow trout (*Oncorhynchus mykiss*) [50,51]. The common carp study generated a new carp genome assembly to analyze gene maintenance following WGD events, given that this species is allotetraploid, and found that all but one of the conserved orthologs were still present; moreover, most ISGs did not undergo additional gene duplication events [50]. On the other hand, the study done on salmonid fishes did not focus on IFN-dependent stimulation of gene expression but rather used both in vivo and in vitro poly(I:C) treatment of head kidney samples as a proxy to generate transcriptomics data [51]. They then mined these transcriptomics datasets to identify whether these zebrafish and tetrapod conserved ISGs [49] were also expressed in other fish species-specific contexts. Here, they found that the core ISG response was overall well conserved between human, zebrafish, rainbow trout, and salmon, given that only eight ISGs had no rainbow trout nor salmon upregulated orthologs [51]. They also identified an additional 100 rainbow trout or salmon poly(I:C)-induced genes which had no ortholog induced by IFNφ1 in zebrafish larvae but did possess a human ortholog. This must be taken with a grain of salt, given that genes upregulated in response to poly(I:C) treatment are not synonymous with ISGs, though a large degree of overlap certainly exists. Notably, these genes included innate immune signaling components (e.g., IRF1 and 2, TLR7, MyD88). They also found that many of the earlier identified zebrafish-specific ISGs, including finTRIM and *btr* family members, were also upregulated in rainbow trout and salmon in response to poly(I:C) treatment [49,51]. In accordance with the diverse environments and evolutionary pressures acting upon Atlantic salmon, both a fresh and saltwater fish, and rainbow trout, an exclusively freshwater fish, many upregulated genes in one species had no orthologs among upregulated genes of the other [51]. This again testifies to the extraordinary adaptive potential of teleost genomes and fast adaptation of their regulatory sequences in transcriptional profiles in response to virus-induced evolutionary pressures.

Finally, an additional study also on Atlantic salmon endeavored to examine the genomic regulatory landscape underlying the antiviral response [52]. Here, fish were stimulated with poly(I:C) before subjecting head kidney cells to a variety of sequencing techniques to identify accessible chromatin (ATAC-seq), transcriptionally active histone marks (ChIP-seq), and induced transcripts (mRNA-seq). The authors then examined the overlap of these markers to identify a putative set of 197 ISGs, wherein their regulatory elements showed both increased chromatin accessibility and the active histone mark H3K27ac in concert with increased gene expression [52]. By cross-referencing previous work, this study identified only 54 genes from this set overlapping with the core set of conserved Atlantic salmon ISG between salmon and human [51]. This set included canonical antiviral genes such as TRIM25, Viperin, IRF7, Mx1, and IFI44. They also performed a transcription factor binding site enrichment analysis on these genes and found the presence of an IRF8 binding site in the regulatory regions of 37 of these 197 genes. IRF8 is one of the transcription factors capable of binding to and driving expression of IFN-stimulated response element (ISRE)-containing promoters. However, in mammals IRF-8 expression is restricted to immune cells [53]; in fact, canonical type I IFN signaling relies primarily on IRF-3, IRF-7, and IRF-9, so it is intriguing that the authors here chose to use IRF-8 as a proxy for ISRE presence. Interestingly enough, the authors found paralogue-specific ISRE enrichment and alterations in chromatin accessibility for key ISGs, likely a consequence of the evolution of distinct regulation following four rounds of WGD events in salmonids [52].

## 3. Antiviral Mechanisms of Conserved ISGs Across Teleosts and Mammals

Given the many conserved ISGs identified between teleosts and mammals, this next section will focus on describing some canonical antiviral effector ISGs and highlight how their mechanisms in fish are either conserved or diverge from their roles in mammalian innate immunity.

### 3.1. IFIT1

The broad-spectrum antiviral IFN-induced protein with tetratricopeptide repeats (IFIT) family of ISGs is conserved across vertebrates [54,55]. The four ISG family members in humans (IFIT1/ISG56, IFIT2, IFIT3, and IFIT5) have been reported to directly inhibit viral replication through diverse mechanisms, including translation inhibition, recognition of unique viral RNA structures, and direct binding to viral proteins [56,57,58,59]. Moreover, IFITs are able to modulate immune signaling. IFIT1 has been reported to demonstrate both positive and negative regulation of the type I IFN response; it both promotes type I IFN expression during alphavirus infection [60] and is able to inhibit IFN production by disrupting interaction of the upstream signaling components STING and MAVS or TBK1 [61].

The IFIT family has expanded beyond its core five human members in zebrafish to at least 10 members [55,62]. Sequence analysis reveals differing number of tetratricopeptide repeat (TPR) motifs within the 10 IFIT proteins, ranging from two to 11 TPR motifs; in contrast, human and murine IFIT genes contain 7–11 TPR motifs [62]. This expansion implies repeated viral interactions and strong selective diversifying pressures; it is possible that individual family members have taken on the contradictory roles of human IFIT1 in regulating the type I IFN response. Many of these IFIT members are upregulated in response to viral infection and have been reported as ISGs [45,46,47,48,49,51,52]. Moreover, despite diversification, some of the most induced IFITs upon viral infection, IFIT12B, IFIT13A, and IFIT17A, were able to protect zebrafish larvae from the spring viraemia of carp virus (SVCV, -ssRNA)-induced mortality [62].

A more detailed exploration of IFIT1 expression and antiviral activity was conducted in the context of the orange-spotted grouper (*Epinephelus coiodes*) [63]. Here, phylogenetic analyses revealed that fish IFIT1s clustered separately from mammal IFIT1s, and that EcIFIT1 expression was significantly upregulated by both DNA and RNA virus infections. EcIFIT1 displayed diffuse cytoplasmic staining. Moreover, overexpression of EcIFIT1 positively regulated the IFN response by inducing the expression of IFN signaling molecules and ISGs (IRF3, IRF7, ISG15, and Mx1) as well as some proinflammatory cytokines (IL-1β, IL-6, and TNF-α). This study also demonstrated that EcIFIT1 overexpression modulated the cell cycle by inhibiting the S/G2 transition to arrest cells in S phase [63].

### 3.2. Mx

Myxovirus resistance proteins (Mx) are dynamin-like GTPases conserved across eukaryotes that exhibit broad, diverse mechanisms of antiviral activity in response to IFN induction [64], though recent evidence suggests that this family predates the dawn of IFN signaling itself [65]. Humans and mice have two Mx genes (MxA and MxB in humans, Mx1 and Mx2 in mice), while this family contains anywhere from one to ten members in different fish species [49,66,67]. Mx family members possess an N-terminal GTPase domain followed by an elongated stalk, which consists of a middle domain and a GTPase effector domain, shared with dynamin, at the C-terminal end. This stalk mediates Mx membrane binding as well as its dimerization and oligomerization into ring-like structures [68,69]. Mammalian Mx antiviral mechanisms vary depending on specific species and viral contexts, but generally depend on their binding to viral components directly to inhibit viral RNA synthesis, prevent capsid uncoating, sequester viral proteins, or block nuclear import [70,71,72,73,74].

Mx is also IFN-inducible and generally antiviral across teleost fish [44,45,46,47,49,50,51,52], though its expansion in some fish species has led to isoform-specific expression, features, and activity. Expression analysis studies of Mx in Atlantic salmon, rainbow trout, and mandarin fish (*Siniperca chuatsi*) have focused on its response to different IFN types, finding specific clusters of Mx genes that respond better to the type II IFN, IFNγ, as opposed to type I [66,67,75]. This may suggest adaptations of some Mx isoforms to work more in organism-wide IFNγ-induced proinflammatory responses, rather than the innate intracellular defenses afforded by the type I IFN response. A genomic analysis of Mx in Indian snow trout (*Schizothorax richardsonii*) and other representative fish species found that fish Mx genes were missing a lysine-rich stretch within the stalk that may determine antiviral specificity and is responsible for membrane interactions in human MxA [69,76,77]. Analysis of Mx in Chinese seabass (*Lateolabrax maculatus*) found that LmMxA has a protective effect against SVCV infection in a dose-dependent manner and may also participate in antibacterial responses against *Vibrio anguillarum* and *Staphylococcus aureus* [78]. Mechanistic studies of Mx in black rockfish (*Sebastes schlegelii*) revealed its ability to inhibit SVCV replication, likely through interfering with viral entry. SsMx was observed to interact strongly with the SVCV glycoprotein [79], which may be required for its antiviral activity and is in line with antiviral mechanistic trends observed in mammalian Mx proteins. On the other hand, the teleost-specific grass carp (*Ctenopharyngodon idella*) Mx isoform, MxG, actually promoted viral replication by negatively regulating MAVS- and STING-mediated antiviral responses [80]. Here, MxG interacted with and promoted both proteasomal-dependent degradation of MAVS and lysosomal-dependent degradation of STING, in addition to inhibiting the activating TBK1-dependent phosphorylation of STING. The GTPase and GTPase effector activity contribute to but are not essential for MxG negative regulatory function [80]. Further investigation of other teleost-specific Mx isoforms may reveal more Mx “traitors” in future.

### 3.3. Nmi and IFP35

The two ISGs N-myc/STAT interactor (Nmi) and IFN-induced protein 35 kDa (IFP35/IFI35) go hand in hand. Indeed, they share conserved tandem Nmi/IFP35 homology domains (NIDs) which mediate their association; both are capable of homodimerization and heterodimerization with one another, as well as with other cellular factors. Both mammalian Nmi and IFP35 encode these tandem NIDs at their C-terminus, but differ in their N-terminal domains: Nmi encodes a coiled-coil (CC) domain while IFP35 encodes an atypical leucine zipper domain incapable of binding DNA [81].

Though mostly known for its role as a transcriptional regulator via interactions with diverse cellular transcription factors [82], Nmi is capable of directly inhibiting replication of the retrovirus prototype foamy virus by sequestering the viral protein Tas in the cytoplasm [83]. In a separate context, Nmi overexpression negatively regulated Sendai virus-induced type I IFN production by promoting proteasome-dependent degradation of IRF7 [84].

Similar roles for IFP35 have been reported. Like Nmi, IFP35 also interacts with transcription factors, associating with B-ATF upon IFN induction to negatively regulate AP-1 transcriptional events [85]. IFP35 also inhibits replication of the related bovine foamy virus by associating with the viral protein Tas, though it does not sequester it as Nmi does [86]. Moreover, IFP35 has been reported to negatively regulate the IFN response in vesicular stomatitis virus infection by targeting the dsRNA sensor RIG-I, both suppressing its activation and promoting its proteasome-dependent degradation [87]. Upon IFN induction, Nmi and IFP35 are upregulated and co-localize in large cytoplasmic punctate, which is thought to have a protective effect in shielding IFP35 from proteasome-mediated degradation [88]. It has alternatively been reported that IFN induction causes IFP35 to move to the nucleus, in keeping with its ability to interact with transcription factors [89].

IFP35 has been identified as a virally induced gene in teleost fish in several transcriptomic and genomic studies [44,46,49,50]. As in mammals, fish IFP35 plays pleiotropic roles in viral replication (Table 1). Whereas overexpression of IFP35 in orange-spotted grouper and rock bream (*Oplegnathus fasciatus*) induces ISG expression and suppresses replication of red-spotted grouper nervous necrosis virus (RGNNV, +ssRNA) and VHSV, IFP35 overexpression in snakehead cells promotes snakehead vesiculovirus (SHVV, -ssRNA) replication [90,91,92]. In mandarin fish, ScIFP35 lacks the N-terminal leucine zipper domain, replaced by an uncharacterized protein family 0242 (UPF0242) domain in tilapia [93], but is still capable of homodimerization [94]. Exogenous ScIFP35 and endogenous ScIFP35 exhibited differing subcellular localization upon poly(I:C) and IFN treatment, with exogenous ScIFP35 forming cytoplasmic punctate only and endogenous ScIFP35 forming both nuclear and cytoplasmic punctate [94]. These data could help explain conflicting reports of mammalian IFP35 localization.

Nmi should also be considered a bona fide teleost ISG. Not only were ISREs identified in both zebrafish and tilapia Nmi promoters, but also infectious spleen and kidney necrosis virus (dsDNA, ISKNV) infection, poly(I:C) treatment, and type I IFN treatment all upregulated its expression in mandarin fish, where it formed cytoplasmic speckles [95,96].

In fact, Nmi and IFP35 negatively regulate the type I IFN response by promoting the degradation of IRF3 and IRF7 and simultaneously protecting each other from degradation [95]. The generation of Nmi and IFP35 domain deletion mutants helped identify essential domains for Nmi-IFP35 interaction, cellular localization, and mutual protection. Nmi structural integrity seems to be important for their ability to localize into cytoplasmic punctate aggregates; deletion of any Nmi domains resulted in a uniform diffuse cytoplasmic distribution for both Nmi and IFP35 and a lowered ability to protect IFP35 from degradation [95]. However, only the Nmi CC domain was required for their interaction; no singular IFP35 domain abolished the Nmi-IFP35 association, though deletion of the IFP35 N-terminal domain reduced the ability of IFP35 to protect Nmi from degradation. Finally, degradation of Nmi, IFP35, and IRF3 and IRF7 seem to be autophagy-dependent, and largely driven by the association of Nmi with the IRFs [95]. It may be that this granular aggregation of the Nmi-IFP35 complex in the cytoplasm is key in regulating IFN signaling and viral infection.

### 3.4. Viperin

Viperin (virus inhibitory protein, endoplasmic reticulum-associated, IFN-inducible; gene name *RSAD2*) is a broad-acting antiviral ISG. Given many decades of research on Viperin, several excellent reviews have been written in recent years detailing its structure and antiviral mechanisms [97,98,99]. Briefly, Viperin consists of three domains: an N-terminal amphipathic helix domain that mediates its membrane associations, a radical S-adenosylmethionine (SAM) domain, and a highly conserved C-terminal domain [100,101]. The radical SAM domain is essential for its antiviral activity against many viruses, allowing Viperin to convert CTP to 3′-deoxy-3′,4′-didehydro-CTP (ddhCTP), which terminates RNA synthesis when incorporated by some, but not all, viral RNA-dependent RNA polymerases [102]. The C-terminus not only helps orient CTP or UTP as substrates for Viperin [103], but also exhibits antiviral activity on its own by enhancing the type I IFN response, antagonizing viral RNA polymerases. Moreover, Viperin is thought to exert additional antiviral activity by activating ubiquitin E3 ligases, which can activate the type I IFN response [104] or promote the degradation of viral proteins via the ubiquitin-proteasome system [105,106].

As a conserved ISG, Viperin is consistently upregulated across diverse teleost fish species in response to IFN and viral infection [45,46,47,48,49,50,51,52,107,108]. Moreover, in keeping with its mammalian counterpart, its overexpression inhibits viral replication against diverse species and viral contexts, blocking GCHV in crucian carp (*Carassius auratus*) [109], rock bream iridovirus (RBIV-C1, dsDNA) in rock bream [110], Singaporean grouper iridovirus (SGIV, dsDNA) replication in orange-spotted grouper and golden pompano (*Trachinotus ovatus*) [111,112], carp edema virus (CEV, dsDNA) in the common carp [113], and VHSV in rockfish and redlip mullet (*Liza haematocheila*) [114,115]. This antiviral activity may be due in part to positive regulation of the IFN response (Table 2). Viperin overexpression increased the expression of IFNc, IRF3, and ISG15 in golden pompano [112]. Moreover, its overexpression also activated IRF3, IRF7, and IFN1 promoters in large yellow croaker (*Larimichthys crocea*) in a manner synergistic with overexpression of IRAK1, the kinase responsible for phosphorylating IRF7 and subsequent type I IFN induction [116]. This IRAK1–Viperin partnership has previously been demonstrated in mammals [117]. In fish, as also in mammals, Viperin consistently localizes to the endoplasmic reticulum [111,114,115].

Viperin-dependent positive regulation of the IFN response and viral protein degradation have been demonstrated in teleost fish. A novel splice variant of Viperin (Viperin_sv1) lacking exon 5, which encodes 11 amino acids close to the catalytic region, was identified in fathead minnow (FHM) cells [119]. Interestingly, Viperin_sv1 but not Viperin was induced only in response to viral infection by SVCV but not poly(I:C) induction, and was able to activate RIG-I, IRF3 and IRF7 signaling cascades, in part by stabilizing RIG-I protein expression, to positively regulate the IFN response [119,120]. This viral-dependent induction of Viperin_sv1 was linked to the P protein of SVCV specifically [120]. Homologs of Viperin within gibel carp, a recurrent polyploid fish, demonstrated mildly divergent antiviral activity in the context of crucian carp herpesvirus (CaHV, dsDNA) infection [122]. While both homologs Viperin A and B were capable of promoting the degradation of CaHV ORF46R, a negative regulator of host IFN production, by suppressing its stabilizing K63-linked polyubiquitination, only Viperin B was able to also promote autophagy-dependent degradation of ORF46R [122]. Further investigation of homolog-specific differences that underlie their divergent antiviral mechanism may provide novel insights into the Viperin antiviral mechanism.

Viperin antiviral activity has also been linked to metabolic regulation. Viperin inhibits cholesterol biosynthesis [123,124], which may negatively impact the egress of enveloped viruses which must bud through cell membranes. Transcriptional analysis in a Viperin KO zebrafish cell line during VHSV infection revealed metabolic changes, namely increased lipid synthesis and reduced generation of reactive oxygen species (ROS) [121]. ROS generation is required for the proper activation and function of immune cells such as macrophages and neutrophils [125]. The *viperin*^-/-^ zebrafish displayed higher neutrophil survival and reduced macrophage recruitment at the site of VHSV infection [121], which may be linked to deficient neutrophil activation and delayed macrophage phagocytosis due to insufficient ROS production [118]. This phenotype of increased macrophage phagocytic activity and induced respiratory burst in response to Viperin overexpression was also observed in large yellow croaker cells [118].

### 3.5. TRIM Ubiquitin E3 Ligases

Ubiquitination is a common post-translational modification with pleiotropic effects [126]. Briefly, ubiquitin is a small, ~7 kDa protein that can be covalently attached to other proteins at lysine residues and linked to itself to generate a polyubiquitin chain. Attachments at specific lysine residues within ubiquitin generate different linkage types (e.g., K48, K63) that generate divergent effects. Free ubiquitin within the cytoplasm must first be activated by E1 enzymes, passed to E2 conjugating enzymes which determine the linkage type, then transferred to E3 ligases that determine substrate specificity and actually ubiquitinate different proteins. E3 ligases have different types of catalytic domains, of which RING E3 ligases make up a large portion [127].

All tripartite motif (TRIM) family members are marked by three conserved domains at the N-terminal domain: a RING E3 ligase domain, 1–2 B-Boxes that may contribute to higher order protein complex formation, and a coiled-coil (CC) domain responsible for TRIM self-association [128]. TRIM family members are sorted into subtypes based on domains present in the C-terminus. Subtype IV (C-IV) in particular is characterized by a PRY/SPRY (B30.2) domain, which mediates protein–protein interactions and is thought to determine substrate specificity [129]. TRIMs carrying this C-terminal domain tend to evolve quickly and duplicate to create multigenic families [130], potentially due to repeated evolutionary pressure from viral interactions [131].

This family of ubiquitin E3 ligases has undergone many expansions and is highly diversified in vertebrates. While the invertebrate fruit fly possesses six *TRIM* genes, and non-jawed vertebrates around 30, humans possess over 70 TRIM family members [128]. The expansion of the TRIM family to 60–80 members in mammals is thought to have coincided with the development of innate immunity, given that many of these newer members serve immune-related functions, being cytokine-induced, antiviral effectors, or immune regulators, particularly of innate immune signaling [129,131].

#### 3.5.1. TRIM25

While over 10 TRIMs have been observed to regulate teleost innate immunity in the context of viral infection [132], not many have also been identified as bona fide fish ISGs. One noteworthy exception to this statement is TRIM25, a well-characterized ISG and antiviral effector [9,133]. TRIM25 carries a C-terminal PRY/SPRY domain and is perhaps best known for its role in positively regulating the IFN response via RIG-I activation through K63-linked polyubiquitination [134]. However, more recent work has focused on its RNA binding activity linked to antiviral mechanisms of inhibition targeting viral translation and ubiquitination of viral nucleoprotein complexes [135,136,137]. TRIM25 also functions as an essential co-factor for the zinc finger antiviral protein (ZAP), for which its ubiquitin E3 ligase activity is essential, though the exact mechanism linking ubiquitination to viral inhibition remains unclear [137,138,139].

TRIM25 is upregulated in response to IFNs and viruses across varied fish species [44,48,49,50,51] and is the most regularly identified TRIM in such transcriptomic studies [132]. Its antiviral function has been demonstrated for fish orthologues of huTRIM25. TRIM25 inhibits SGIV replication in orange-spotted grouper [140], RGNNV in zebrafish [141], and SVCV in common carp [142]. Though its antiviral mechanisms have yet to be thoroughly characterized in fish, fish TRIM25 likely operates along the same lines as its mammalian counterpart. Its RING domain is essential for its antiviral activity [140], and it positively regulates the IFN response by mediating K63-linked polyubiquitination of RIG-I, thus activating RIG-I signaling [141,142]. A solitary report of TRIM25 as a negative regulator of the IFN response in black carp (*Mylopharyngodon piceus*) may represent an isolated, species-specific adaptation of TRIM25. Two studies of poly(I:C) induction of TRIM25 isoforms or splice variants revealed both differential expression in tissues and subcellular localization [143,144]; these may represent early stages of the adaptation of TRIM25 to different roles in the innate immune response.

Specific roles for direct interactions between TRIM25 and viral proteins in fish and antiviral translation inhibition have yet to be identified. This may be due to the lack of a fish ortholog for ZAP, TRIM25’s partner in viral inhibition, which is thought to have emerged alongside the advent of tetrapods [145]. However, a “ZAP-like” gene was identified in common carp [146]. This ZAP-like sequence possesses three WWE domains and none of the zinc fingers after which ZAP is named, so it may be a bit of an overreach to consider it a ZAP-like protein. Still, this ZAP-like protein may exhibit some antiviral activity, as it is upregulated in response to SVCV challenge in vivo and its overexpression diminished the SVCV-induced cytopathic effect in vitro [146].

#### 3.5.2. Fish-Specific finTRIMs

One possible reason that TRIMs have not gained as much traction as ISGs in fish is because their roles would prove redundant in light of the teleost-specific TRIM expansions, namely the fish novel TRIMs (finTRIMs; gene name *ftr*), which are most closely related to TRIM16 or TRIM25, the TRIM39-derived *bloodthirsty*-related (*btr*) family, and TRIM35-related proteins [132,147,148]. TRIM16, TRIM25, and TRIM39 all belong to the quickly evolving C-IV subtype of TRIMs which possess a PRY/SPRY domain at the C-terminal end [148]. In zebrafish, finTRIMs include over 70 members, *btr* genes over 30, and TRIM35 over 30 as well [147,148]. Examination of finTRIMs across puffer fish, stickleback, and zebrafish found that only three FTR genes are evolutionarily conserved across fish species based on B30.2 phylogenetic analysis and regional synteny analyses [147]. Moreover, FTRs within the rapidly evolving group of finTRIMs displayed signs of strong diversifying selection within their PRY/SPRY domain [147]. This provides strong evidence of repeated virus–finTRIM interactions and supports their role as antiviral effectors, potentially as virus recognition or restriction factors.

Unlike their bona fide fish TRIM orthologues, many finTRIMs are induced in response to viral infection and IFN signaling and primarily exhibit the regulatory roles of the IFN response [47,49,51,149,150,151,152,153,154,155,156,157,158]. This is analogous to the role TRIMs play in mammalian innate immunity [129]. However, matters are complicated by fish-specific expansions of these TRIM families, such that finTRIMs lack conservation across species. To account for this, research groups across varied fish species have taken to naming FTR genes in order of their identification and characterization, without attention to any shared features. It would be erroneous to assume “FTR01” in one fish species as orthologous to “FTR01” in another fish species [153]. For the sake of consistency, this review will refer to all finTRIM proteins with the following nomenclature: a two-lettered species abbreviation followed by “FTR” and the gene number used in the study. Both DrFTR36 and DrFTR67 inhibited SVCV replication in zebrafish via positive regulation of the IFN response in a RING domain-dependent manner, though no direct ubiquitination targets were identified [151,152]. On the other hand, negative regulation of the IFN response by finTRIMs usually involved degradation of an IFN signaling component [153,154,155,156,157,158]. In crucian carp, CaFTR1 negatively regulates the IFN response in a pleiotropic manner: not only does it mediate autophagosome-dependent degradation of TBK1 in an ubiquitin ligase-dependent manner, it also enhanced decay of both IRF7 and STING mRNAs [153,154,155]. EcFTR82 negatively regulated the IFN response in the context of SGIV and RGNNV infection [156]. Negative regulation of the IFN response by DrFTR33 in the context of SVCV replication in zebrafish may be mediated through its interaction with MDA5 or MAVS, though the direct impact of this association remains unclear [157]. EcFTR14 targets TRAF6, an indirect activator of NF-κB signaling [159], for proteasomal-dependent degradation to negatively regulate the IFN response in the context of RGNNV infection in grouper [158]. All in all, this consistent theme of E3 ligase-dependent activity in species-specific finTRIM regulation of the innate immune response support the hypothesis that finTRIMs in teleost fish repeatedly diversified in order to regulate an increasingly complex immune system [129].

### 3.6. ISGylation

ISG15 is a conserved member of the ubiquitin family and one of the strongest and most rapidly induced ISGs [160,161]. It is comprised of two ubiquitin-like (UBL) domains linked by a hinge region and is conjugated to its targets at a C-terminal LRLRGG motif through ISGylation, a ubiquitination-like three-step process [162]. Briefly, free ISG15 is activated by UBE1L, passed to an E2 conjugating enzyme, and finally to an E3 ligases which determines substrate specificity. Here, several ubiquitin E3 ligases moonlight as ISG ligases, namely HERC5 (human) or HERC6 (mouse) and TRIM25 [160]. Both host and viral proteins may be ISGylated to disrupt viral replication by interfering with essential protein–protein interactions, nucleocapsid assembly, and viral egress [160]. ISG15 can also be secreted and exhibits cytokine activities to modulate the immune response, typically by enhancing IFN-γ secretion by NK cells and T cells [161].

#### 3.6.1. ISG15

Unsurprisingly, ISG15 is strongly induced in teleost fish in response to varied viral infections across zebrafish, salmon, grass carp, sea bass (*Lates calcarifer*), turbot (*Scophthalamus maximus*), and yellow croaker [44,45,47,48,49,50,163,164,165,166,167]. ISG15 has not been subject to extensive gene duplication, with most species only possessing one homolog, suggesting that its antiviral mechanisms have largely been conserved. As in mammals, ISG15 was demonstrated to possess cytokine-like activity in European sea bass [166]. Here, medium from ISG15 producing cells was protective against RGNNV infection in a dose-dependent manner. Direct incubation of cells with recombinant ISG15 also protected against RGNNV infection. This protective effect may be mediated by positive regulation of the type I IFN response, as cells exposed to ISG15 exhibited upregulation of the classical ISG Mx [166].

Even when ISG15 has undergone additional gene duplication, as within Atlantic salmon, it seems likely that most salmon ISG15 copies do not play a prominent antiviral role. Though Atlantic salmon have two copies of ISG15 on chr. 20 and four copies on chr. 24, only one gene within each region was upregulated in response to infectious salmon anemia virus (ISAV, -ssRNA) infection [167]. However, ISG15 amino acid residues have diverged between species. Human ISG15 possesses a singular, conserved cysteine residue in the hinge region between the UBL domains which is thought to facilitate ISGylation by participating in a disulfide bridge to substrate proteins [168]. Curiously enough, fish homologs of ISG15 have replaced this hinge cysteine residue by proline and instead either possess other non-conserved cysteine residues throughout the protein or none at all [165]. Still, high conservation of the C-terminal LRLRGG motif among fish ISG15 homologs suggests that they still participate in ISGylation as means of inhibiting viral replication [165].

#### 3.6.2. HERCs as ISG15 Ligases

Indeed, transcriptomic analyses of an Atlantic salmon cell line in response to infectious salmon anemia virus (ISAV) found not only strong upregulation of ISG15 but also many ISG15-associated proteins, including the ISG15 ligases TRIM25 and teleost-specific HERC7 and HERC9, the ISG15 interactor and ISGylated cellular sensor RNF213, and the ISG15-specific deubiquitinase USP18 [167]. Upregulation of all these ISG15-associated genes strongly suggests a prominent role for ISGylation in the teleost innate antiviral response. Moreover, both RNF213 and HERC5- and HERC6-related proteins were also identified as virally induced genes or ISGs in zebrafish, salmon, and grass carp [45,46,49,50,51,52].

Though it was originally thought that salmon possessed no direct ortholog for HERC5 or HERC6, the primary mammalian ISG15 ligases, and instead possess the teleost-specific HERC7 and HERC9, regional synteny analyses reveal that HERC7 and HERC9 are located in the analogous location to HERC5 and HERC6: sandwiched between the genes ABCG2 and PPM1K on one side and FAM13A on the other [167]. This strongly suggests that teleost HERC7 and HERC9 have evolved from mammalian HERC5 and HERC6 counterparts, which provides additional evidence bolstering the role of ISGylation in teleost antiviral defenses. HERC7 has undergone moderate family expansion in teleost, with four members in zebrafish and two in salmon [167,169]. Studies in zebrafish and crucian carp have validated HERC7 as an ISG that negatively regulates the type I IFN response, though through divergent mechanisms [169,170]. In zebrafish, studies of HERC7c found that it only retained ubiquitin ligase activity and targets IFN signaling components STING, MAVS, and IRF7 for protein degradation [169]. On the other hand, crucian carp HERC7 was capable of both ISGylation and ubiquitination, targeting MAVS and STING for proteasome degradation on one hand and inducing IRF7 mRNA decay on the other. Interestingly, crucian carp HERC7 ubiquitin ligase activity was not required for its immunomodulatory activities [170]. Though also induced in response to viral infection, it remains to be seen whether the other zebrafish HERC7 family members (HERC7a, b, and d) also modulate the immune response and/or retain ISGylation catalytic activity, and if so, whether ISGylation is required for their participation in the type I IFN response.

## 4. Conclusions and Future Perspectives

While much progress has been made in identifying virally or poly(I:C)-induced genes in teleost fish, and moderate progress in teasing out the specific antiviral mechanisms of ISGs, most ISG-related studies are still restricted to expressional analyses over functional characterizations. Moreover, the field currently suffers from a lack of truly robust screening for or identification of ISGs. While a number of transcriptomic studies purport to have identified ISGs in a variety of teleost species, in reality most have either relied on an interferon substitute, e.g., poly(I:C) stimulation or viral infection, or performed genome or transcriptome annotations with an eye to identifying orthologs of zebrafish ISGs identified by Levraud and colleagues in their pioneering work [49]. Though the former strategy seems like a plausible shortcut, given that both poly(I:C) and viral infection stimulate the first arm of IFN signaling, producing IFNs which then stimulate production of ISGs, it runs the risk of identifying genes that are stimulated by the first arm directly and are not true ISGs. Such was the case for A20 in teleost fish, wherein transfection of constitutively active RIG-I upregulated its expression but IFN treatment did not [171].

Work remains to be done in conducting screens that rely on pure IFN stimulation or test for IFN receptor dependence in virally induced genes in order to compile a comprehensive ISG library for teleost species. Following this compilation, characterizing these teleost ISGs as regulators of the IFN response or as antiviral effectors remains an outstanding task [172]. Doing so may well reveal potential drug targets to enhance immunity in fish or enable the design of novel ISGs to be repurposed for human health. For example, exploring the antiviral nature of the finTRIM family may identify novel mutations within the PRY/SPRY domain that can be applied towards its mammalian TRIM counterparts within C-IV, such as the antiviral ISGs TRIM22 or TRIM25. Moreover, teleost ISG identification and characterization could play the dual role of serving as biomarkers for the diagnosis of viral and inflammatory diseases in fish, especially as aquaculture practices continue to burgeon in importance. Miller and colleagues have made great strides in this area in their multi-study analysis of differentially expressed genes in Atlantic salmon to begin identifying biomarkers of viral disease development in wild migrating salmon [46].

Teleost ISG characterization represents an especially monumental task when one considers the depth of subfunctionalization and specialization that has occurred in teleost species. As previously discussed, species-specific finTRIM diversification represents just one example of how complex the road to illuminating teleost ISG functions is. One might envision replicating the work done by Levraud and colleagues in identifying ancestral ISGs not just in zebrafish, but in other well-characterized, economically relevant species that represent a wider phylogenetic spread within teleost species such as Atlantic salmon or Asian seabass [49]. Comparing extant ISGs within a spread of teleost species would enable identification of conserved teleost-specific ISGs.

Another interesting avenue of research could be investigating the role of stress granules in fish antiviral responses. Many of the cytoplasmic ISGs, especially finTRIMs, displayed punctate distribution in the cytoplasm when examined with immunofluorescence [95,150,152,153,158], which is characteristic of stress granule staining. In fact, a screening of ISG15 interactors via yeast-2 hybrid interaction analysis yielded a number of translation-related proteins [173]. Stress granules are LLPS structures that collect paused translational machinery and have been shown to exhibit an antiviral effect [174,175]. It would be interesting to perform protein interaction studies to see if these cytoplasmic punctate-distributed fish ISGs also localize to stress granules. Still, it must be noted that this punctate distribution has only been observed under ectopic overexpression studies of these ISGs, and it remains to be seen whether the endogenously expressed ISGs would display the same punctate distribution in the context of a viral infection.

Cell-type specific studies and in vivo antiviral mechanistic studies may prove fruitful. While it is understandable that most initial characterization studies of antiviral mechanisms take place within cell cultures, given that cells are much more amenable to genetic manipulation and analysis, doing so may overlook cell-type specific effects of individual ISGs. For example, as described earlier, Viperin exerted a macrophage-specific antiviral effect within the large yellow croaker [118]. This would have been missed in a simple in vitro study working with one’s favorite cell line.

Teleost ISGs with all of their species-specific intricacies and nuances hold great promise of unearthed potential. One might even say that a whole ocean’s worth of informational treasure remains to be brought up from the depths.

## Figures and Tables

**Table 1:** Pleiotropic effects of IFP35 on viral replication in human and teleost fish. Species refers to the genetic source of IFP35 used in the study.

Species	Cell Context	Virus	Effect on Viral Replication	Direct or Indirect ^1^	Mechanism	Ref.
Human	HEK293T	BFV	Antiviral	Direct	Interacts with viral protein Tas	[86]
Human	HeLa, HEK293	VSV	Proviral	Indirect	Suppresses RIG-I activation and targets it for proteasome-dependent degradation	[87]
Orange-spotted grouper	GS	RGNNV	Antiviral	Indirect	Positively regulates IFN response	[92]
Rock bream	FHM	VHSV	Antiviral	Indirect	Positively regulates IFN response	[91]
Striped snakehead	SSN-1	SHVV	Proviral	—	—	[90]
Chinese perch	MFF-1	SCRV	Proviral	Indirect	Negatively regulates IFN response by promoting IRF3 and IRF7 degradation	[95]

^1^ A horizontal line is provided when the mechanism of viral replication was not elucidated in the study.

**Table 2:** Mechanisms of Viperin antiviral activity in teleost fish. Species refers to the genetic source of Viperin used in the study.

Species	Cell Context ^1^	Virus	Direct or Indirect	Mechanism	Ref.
Golden pompano	TOPF	SGIV	Indirect	Positively regulates IFN response	[112]
Large yellow croaker	LCM10	LYCIV	Indirect	Positively regulates IFN response; increased macrophage phagocytic activity and induced respiratory burst	[118]
Fathead minnow	FHM	SVCV	Indirect	Positively regulates IFN response by stabilizing RIG-I expression	[119,120]
Zebrafish	—	VHSV	Indirect	ROS production linked to neutrophil activation and macrophage phagocytosis	[121]
Gibel carp	CAB	CaHV	Direct	Promotes degradation of ORF46R, a negative regulator of IFN response	[122]

^1^ A horizontal line is provided when no cell lines were used for elucidation of antiviral mechanisms.

## Data Availability

No new data were created or analyzed in this study.
